# 3-Bromopyruvate-mediated MCT1-dependent metabolic perturbation sensitizes triple negative breast cancer cells to ionizing radiation

**DOI:** 10.1186/s40170-021-00273-6

**Published:** 2021-10-14

**Authors:** Irini Skaripa-Koukelli, David Hauton, John Walsby-Tickle, Eloïse Thomas, Joshua Owen, Abirami Lakshminarayanan, Sarah Able, James McCullagh, Robert C. Carlisle, Katherine A. Vallis

**Affiliations:** 1 Department of Engineering Science, Institute of Biomedical Engineering, University of Oxford, Old Road Campus Research Building, Oxford, OX3 7DQ UK; 2 Department of Oncology, Oxford Institute for Radiation Oncology, University of Oxford, Old Road Campus Research Building, Off Roosevelt Drive, Oxford, OX3 7DQ UK; 3 Department of Chemistry, Chemistry Research Laboratory, University of Oxford, Oxford, OX1 3TA UK

**Keywords:** Triple-negative breast cancer, 3-Bromopyruvate, Metabolism, Ionizing radiation

## Abstract

**Background:**

Triple negative breast cancer (TNBC) poses a serious clinical challenge as it is an aggressive form of the disease that lacks estrogen receptor, progesterone receptor, and ERBB2 (formerly HER2) gene amplification, which limits the treatment options. The Warburg phenotype of upregulated glycolysis in the presence of oxygen has been shown to be prevalent in TNBC. Elevated glycolysis satisfies the energy requirements of cancer cells, contributes to resistance to treatment by maintaining redox homeostasis and generating nucleotide precursors required for cell proliferation and DNA repair. Expression of the monocarboxylate transporter 1 (MCT1), which is responsible for the bidirectional transport of lactate, correlates with an aggressive phenotype and poor outcome in several cancer types, including breast cancer. In this study, 3-bromopyruvate (3BP), a lactate/pyruvate analog, was used to selectively target TNBC cells that express MCT1.

**Methods:**

The cytotoxicity of 3BP was tested in MTT assays using human TNBC cell lines: BT20 (MCT1^+^/MCT4^−^), MDA-MB-23 (MCT1^−^/MCT4^+^), and BT20 in which MCT1 was knocked down (siMCT1-BT20). The metabolite profile of 3BP-treated and 3BP-untreated cells was investigated using LC-MS/MS. The extracellular acidification rate (ECAR) and oxygen consumption rate (OCR) of BT20 and MDA-MB-231 cells treated with 3BP were measured using a Seahorse XF96 extracellular flux analyzer. The impact of ionizing radiation on cell survival, alone or in combination with 3BP pre-treatment, was evaluated using clonogenic assays.

**Results:**

Metabolomic analyses showed that 3BP causes inhibition of glycolysis, disturbance of redox homeostasis, decreased nucleotide synthesis, and was accompanied by a reduction in medium acidification. In addition, 3BP potentiated the cytotoxic effect of ionizing radiation, a treatment that is frequently used in the management of TNBC.

**Conclusions:**

Overall, MCT1-mediated metabolic perturbation in combination with radiotherapy is shown to be a promising strategy for the treatment of glycolytic tumors such as TNBC, overcoming the selectivity challenges of targeting glycolysis with glucose analogs.

**Supplementary Information:**

The online version contains supplementary material available at 10.1186/s40170-021-00273-6.

## Background

Breast cancer is the most common cancer globally and the second most frequent cause of cancer-related mortality in women [1]. Triple-negative breast cancer (TNBC) is a subtype of breast cancer characterized by the absence of estrogen receptor (ER), progesterone receptor (PR) expression, and of epidermal growth factor receptor 2 (ERBB2) gene amplification. Most TNBC tumors belong to the basal-like subtype (50-80%), and conversely, more than 90% of basal-like tumors are triple-negative [2, 3]. TNBC has a poor prognosis, with a 5-year breast cancer-specific survival rate of 85% for stage I tumors (compared to 99% and 95% for hormone or ERBB2-positive groups, respectively). In metastatic TNBC, the median survival falls to 1 year, compared to 4-5 years for the hormone and ERBB2-positive groups [4]. An encouraging recent development is the approval of poly(ADP-ribose) polymerase inhibitors (PARPi), a family of targeted therapeutic small molecules, which are effective in TNBC patients with BRCA 1/2 germline mutations, comprising 10-15% of TNBC cases [4, 5]. However, for the majority of patients with TNBC, while surgery and radiation therapy lower the risk of loco-regional recurrence, chemotherapy with cytotoxic agents remains the major systemic treatment option [4]. The lack of molecular targets together with the aggressive nature of these tumors limits treatment success. Therefore, novel therapeutic approaches selectively targeting the characteristics of cancer cells and those of the tumor microenvironment are urgently needed to improve clinical outcomes.

Altered metabolism is a well-recognized hallmark of cancer [6]. Cancer cells preferentially upregulate glycolysis, even under aerobic conditions, a phenomenon termed the “Warburg effect.” Although the extent to which different cancer cells utilize glycolysis and oxidative phosphorylation (OXPHOS) varies, the Warburg phenotype has been shown to predominate in TNBC tumors [7]. Glycolytic metabolism is known to correlate with chemo- and radioresistance [8, 9]. Bhatt et al. found that transient pharmacological stimulation of glycolysis conferred radioresistance to both malignant and non-malignant cells in vitro [8]. Consistent with these findings, Rashmi et al. showed that the glycolysis inhibitor 2-deoxy-glucose (2DG) radiosensitizes glycolytic cervical cancer cells (CaSki), an effect that was potentiated by simultaneous inhibition of glutathione (GSH) and thioredoxin metabolism [10]. This observation suggests that the enzymes and transporters that support glycolysis are suitable targets for the treatment of TNBC, and several compounds of this type are in preclinical development [11]. However, the lack of selectivity of currently available anti-glycolytic compounds such as 2DG limits this approach [12].

Plasma membrane transporters are involved in the uptake of nutrients, regulation of intracellular pH and efflux of the by-products of metabolism. They include transporters for glucose, amino-acids, and monocarboxylates. Monocarboxylate transporters (MCT) are transmembrane proteins encoded by the SLC16A gene family, consisting of 14 isoforms. Only MCT1, 2, 3, and 4 are known to catalyze the proton-linked transport of lactate, pyruvate, and other monocarboxylates. MCT1 is expressed in multiple tissues, whereas MCT4 expression is mainly associated with tissues that rely on glycolysis, such as white muscle fibers, astrocytes, and leukocytes [13]. Although MCT-mediated transport is bidirectional, MCT4 is primarily involved in efflux of lactate while MCT1 is associated with both influx and efflux [13, 14]. Upregulation of MCT1 has been shown in several tumor types and is correlated with poor outcome in endometrial, renal, pancreatic, and lung malignancies [15]. In breast cancer, high expression of MCT1 is associated with the triple-negative [16] and basal-like phenotype [17], elevated glycolysis [18, 19], and poor outcome in TNBC patients [16], making it of great interest as a therapeutic target.

MCT-mediated lactate efflux provides several advantages to cancer cells, other than maintenance of the glycolytic flux. Lactate reduces the pH of the tumor microenvironment, dampens the immune response, promotes angiogenesis and metastasis, and alters the metabolism of stromal cells that support tumor growth [20]. Therefore, reducing lactate concentration in the tumor microenvironment could reverse these effects, making MCT inhibition an attractive strategy. Efforts to target MCT1 have focused on small molecule inhibitors. Examples are lonidamine, quercetin, and α-cyano-4-hydroxycinnamic acid (CHC), which competitively bind to and inhibit the transporter [14, 21]. These molecules have been tested in pre-clinical studies but lack specificity for MCT1. A group of specific MCT1/2 inhibitors, which are reported to bind to the transporter intracellularly [22], have been developed by AstraZeneca [23] and one of them, AZD3965, is currently under clinical evaluation for advanced tumors (NCT01791595). Unfortunately, inhibition of a single isoform may be insufficient to suppress lactate efflux as cancer cells commonly express more than one isoform, which could confer resistance. Further, although involvement of MCT1 has been implicated in invasion and migration, pharmacological inhibition of the transporter has not consistently been shown to prevent these processes [21, 24, 25]. Another approach is to exploit MCT1-mediated transport of toxic molecules, such as 3-bromopyruvate (3BP), selectively into cancer cells. Birsoy et al. linked IC_50_ values of 3BP in 15 cell lines with transcriptome-wide mRNA expression data, and found that MCT1 was a major determinant of sensitivity to the compound [26]. 3BP has proven to be a highly potent anti-cancer compound: its cytotoxicity attributed primarily to effects on glycolysis, although disruption of mitochondrial metabolism and redox equilibrium, have also been identified in cells and in cell-free conditions [27]. In the glycolytic pathway, hexokinase II (HX-2) and glyceraldehyde phosphate dehydrogenase (GAPDH) in particular have been identified as targets [28–30].

Given the multiplicity of 3BP mechanisms of action, its effects are best evaluated using an unbiased metabolomics approach. Although chromatographic separation coupled with mass spectrometry (MS) is an efficient and sensitive method allowing comprehensive analysis of metabolic states, technical challenges arise in the analysis of metabolic changes in biological systems such as cell lysates. Specifically, small organic acids, which are found in their anionic state under physiological conditions, constitute the majority of the metabolites involved in the central carbon metabolism. Additionally, loss of phosphate groups from multiply phosphorylated metabolites (e.g., ATP, GTP) upon electrospray ionization can skew the metabolite distribution of the sample toward lower phosphorylation states. In response to these challenges, we recently developed and validated an anion-exchange chromatography coupled to high-resolution orbitrap MS (IC-MS) protocol, offering coverage of the central carbon metabolism and linear, reproducible loss of phosphates which does not alter the sample characteristics [31]. In this study, we used this IC-MS method to elucidate, in an unbiased and comprehensive way, the effects of 3BP on the metabolism of TNBC cells.

Radiation is very frequently used as an adjuvant treatment after breast cancer surgery, substantially reducing the local recurrence rate in patients with TNBC [32]. Whether TNBC sensitivity to ionizing radiation differs from other breast cancer subgroups remains unclear. Despite the application of surgery and radiotherapy, TNBC is characterized by an increased loco-regional recurrence rate [32–34]. We hypothesized that the metabolic perturbation caused by 3BP, in particular its effects on glycolysis, redox homeostasis, and nucleotide synthesis, could potentiate the cytotoxic effects of ionizing radiation with selectivity toward cells expressing MCT1.

Overall, we aimed to investigate the potential of 3BP, a small molecule “metabolic poison,” to selectively target TNBC cells by exploiting the overexpression of MCT1 combined with the Warburg phenotype. We show that 3BP treatment leads to MCT1-dependent metabolic catastrophe resulting in oxidative stress, depletion of the nucleotide pool, decreased glycolysis and lactate excretion, and ultimately resulting in increased cytotoxicity and radiosensitivity.

## Methods

### Cell lines

The Broad Institute Cancer Cell Line Encyclopedia (CCLE) was used to compare the expression of MCT isoforms in 57 breast cancer cell lines. RNA expression (RNAseq) data, expressed as log_2_ (fold change) (FC) were plotted using Prism7 (GraphPad, CA, USA). Co-expression of MCT1 and MCT2 (high affinity isoform) or MCT4 (low affinity isoform) was evaluated. BT20, MDA-MB-231, MDA-MB-468, and BT549 cells were purchased from the American Type Culture Collection (ATCC) and cultured in Dulbecco’s Modified Eagle Medium (DMEM), except BT549 which was cultured in RPMI, supplemented with 10% fetal bovine serum (FBS) and 1% penicillin/streptomycin/l-glutamine. Cell lines were maintained at 37 °C in a humidified, 5% CO_2_ atmosphere (HeraCell™ 150 incubator, Thermo Fisher Scientific, Waltham, USA) and sub-cultured using trypsin-EDTA 0.05% solution. Cells were tested monthly and found to be mycoplasma-free. Cells were discarded at passage number 25 or lower, counting from the original ATCC stock. MDA-MB-231, MDA-MB-468, and BT20 cells were authenticated by ATCC.

### Western blot analysis

Cells were dissociated from the culture flask with trypsin, were pelleted, were washed with cold phosphate-buffered saline (PBS) solution, and were lysed with RadioImmunoprecipitation Assay (RIPA) lysis buffer supplemented with a protease inhibitor cocktail (Thermo Fisher Scientific, MA, USA). Protein content of lysates was determined by BCA assay (Thermo Fisher Scientific, MA, USA). Samples were prepared in lithium dodecyl sulfate (LDS) loading buffer supplemented with sample reducing agent (NuPAGE™, Thermo Fisher Scientific, Waltham, USA), heated at 70 °C for 10 min, resolved by NuPAGE® 4–12% Bis-Tris gels and LDS-PAGE, and transferred onto a nitrocellulose membrane. Nitrocellulose membranes were blocked for 1 h at room temperature with 5% w/v dry, non-fat milk in PBS-Tween 20 0.05% v/v solution. Membranes were probed with antibodies against MCT1 (sc-365501, 1:200 dilution, Santa Cruz Biotechnology, CA, USA) or MCT4 (sc-50329, 1:500 dilution, Santa Cruz Biotechnology, CA, USA) in 0.5% w/v milk in PBS (4 °C, overnight incubation). β-Actin was used as a loading control for all blots (ab8227, Abcam, Cambridge, UK, 1:1000 dilution). Reactions were visualized using a suitable secondary antibody (1:2000 dilution) conjugated with horseradish peroxidase (HRP). Pierce ECL (Thermo Fisher Scientific, MA, USA) or WesternSurePREMIUM (LI-COR Biosciences, Nebraska, USA) chemiluminescent substrates and a digital scanner (C-Digit Blot Scanner, LI-COR Biosciences, Nebraska, USA) were used to visualize protein expression. Full membrane images for all Western blots are shown in Figure S1.

### MTT assay

Cells were seeded in 48 well plates (5 × 10^4^ cells/well), were allowed to adhere, and then were treated with 0–300 μM freshly prepared 3BP solution for 1 or 24 h. After treatment, the medium was replaced with fresh serum-free medium supplemented with thiazolyl blue tetrazolium bromide, 0.5 mg/mL. Following 45 min incubation at 37 °C, the medium was removed and formazan crystals dissolved in dimethyl sulfoxide (DMSO). Absorbance at 540 nm (with reference at 630 nm) was quantified using a plate reader (Infinite 200 Pro, TECAN, Switzerland). The reading (a measure of viability) for treated cells was compared to that of control cells and expressed as % metabolic activity mean value (four replicates).

### Trypan blue exclusion viability assay

Cells were seeded at 4 × 10^5^ cells/well in 6-well plates and allowed to adhere overnight. The following day, cells were incubated with fresh medium containing 20, 100, or 150 μM 3BP for 1 or 24 h. At the end of the incubation period, the 3BP-containing medium was removed; cells were trypsinized, were pelleted, and were resuspended in growth medium. The cell suspension was mixed at a 1:1 ratio with 0.4% w/v trypan blue solution (Gibco), and viability assessed using the Countess cell counting system (Invitrogen), as per manufacturer’s instructions. Results were reported based on two counts of 4 replicates.

### Transfection with siRNA for MCT1 knockdown

Cells were seeded at 4 × 10^5^ cells/well (approximately 80% confluency for BT20 cells) in 6-well plates. Cells were incubated overnight with siRNA against MCT1 (sc-37235) or control siRNA (sc-37007) (Santa Cruz Biotechnology, CA, USA). For transfection, siRNA solution (100 nM final concentration) was mixed with transfection reagent solution (sc-29528) in serum-free transfection medium (sc-36868), followed by 40 min incubation at room temperature (Santa Cruz Biotechnology, CA, USA). The mix was then diluted with transfection medium to the final siRNA concentration (100 nM) and 1 mL was added to each well. After 6 h incubation, 1 mL of 20% FBS-containing DMEM was added to each well. The transfection medium was replaced the following day and the transfected cells were used for experiments. Transfection efficiency was assessed by Western blot analysis, as described above.

### Metabolomics

#### Sample preparation

Six replicates of each sample were prepared. Fresh medium was added to cells at 80% confluency. The following day, cells were treated with 100 μM 3BP in complete DMEM for 5, 15, 30 or 60 min. For siRNA-transfected samples, cells were grown and transfected as described above, and treated with 3BP 1 day following transfection. After treatment with 3BP, medium was removed and the cells were washed twice with ice-cold PBS. The metabolites were extracted with ice-cold 80% v/v methanol (HPLC grade). The resulting lysates were centrifuged at 20,913×*g* for 30 min at 4 °C. Supernatants were collected and double-strand DNA (dsDNA) concentration was determined using NanoDrop™ (Thermo Fisher Scientific, Waltham, USA). Soluble protein and nucleic acids were removed by centrifugal filtration at 13,000×*g* for 30 min at 4 °C through a pre-washed 10 kDa molecular weight cut-off filter (Amicon Ultra, Millipore). Samples were normalized to approximately 10 ng/μL dsDNA with 80% v/v methanol, transferred into total recovery HPLC vials, and stored at −80 °C for later LC-MS analysis.

#### Analysis of metabolite extracts with LC-MS/MS

Each sample was analyzed using three separate LC-MS/MS methods using two different LC systems (Thermo Scientific ICS-5000+ ion chromatography and Thermo Ultimate 3000). Each was coupled directly to a Q-Exactive HF Hybrid Quadrupole-Orbitrap mass spectrometer with a HESI II electrospray ionization source (Thermo Scientific, San Jose, CA). Full details for each method are provided in the “Supplementary information” section (Supplementary Methods). Briefly, for method 1, anion-exchange chromatography coupled with mass spectrometry (IC-MS/MS) was performed as recently published [31]. Reversed phase C18 column analysis was performed for both methods 2 and 3. For method 2, the samples were used underivatized (same as in method 1) [31], while for method 3, samples were derivatized prior to analysis using a modified version of the Waters AccQ-Tag method designed for amino-acid analysis [35].

#### Data analysis

Raw data files were processed using Progenesis QI (Waters, Elstree, UK). This process included alignment of retention times, peak picking by identification of the presence of natural abundance isotope peaks, characterizing multiple adduct forms and identification of metabolites using an in-house database. Retention times, accurate mass values, relative isotope abundances, and fragmentation patterns were compared between authentic standards and the samples measured. Identifications were accepted only when the following criteria were met: < 5 ppm differences between measured and theoretical mass (based on chemical formula), < 30 s differences between authentic standard and analyte retention times, isotope peak abundance measurements for analytes were > 90% matched to the theoretical value generated from the chemical formula. Where measured, fragmentation patterns were matched to at least the base peak and two additional peak matches in the MS/MS spectrum to within 12 ppm. The top 10 data directed fragmentation method was not always able to provide fragment ions for all ions measured in the MS 1 spectrum. After peak identification, fold change (FC) was calculated within each cell group. For fold change calculation, the following formula was used: log_2_[FC]=log_2_[final]-log_2_[control], where [final] is the normalized abundance at the time point of interest and [control] is the average abundance of the untreated control of this cell sample. Where measurements from different batches were to be compared, the average abundance value of a random selection of metabolites (“QC”) was compared between the two batches (QC ratio). Then, the values of interest were normalized between different batches.

### Bioenergetics

The extracellular acidification rate (ECAR) and oxygen consumption rate (OCR) of BT20 and MDA-MB-231 cells treated with 3BP were determined using a Seahorse XF96 extracellular flux analyzer (Agilent, CA, USA). The day before the experiment, cells were seeded into Seahorse cell culture 96-well plates (20,000 cells/well in 100 μL complete DMEM). Cells were incubated at room temperature (RT) for 30-60 min and then incubated at 37 °C and 5% CO_2_ and allowed to adhere overnight. On the day of the experiment, each well was topped up to 200 μL with complete DMEM +/− 3BP to give a final concentration of 0, 20, or 100 μM. Plates were incubated for 1 h (20 and 100 μM) or overnight (20 μM only). Prior to measurement, growth medium was replaced with measurement (unbuffered) DMEM complemented with glutamine and glucose, as per manufacturer’s instructions. For the ECAR and OCR measurements, the sensor cartridge and cell plate were equilibrated and calibrated according to the manufacturer’s instructions using Seahorse-specific reagents (Agilent Technologies, CA, USA). For the time course experiments, 3BP solution in assay medium (10× final concentration) was loaded into the sensor cartridge to give a final concentration of 20 or 100 μM in the well. The instrument was set to acquire consecutive measurements for 1 h with mixing in between. For the time course, 3BP was injected after acquiring one measurement as baseline ECAR and OCR.

### Clonogenic survival assay

BT20 cells (80-90% confluency) were incubated with 3BP (50, 100, or 150 μM) or fresh medium for 1 h. The medium was replaced with fresh medium and the cells were irradiated using a cesium-137 (^137^Cs) gamma irradiator (dose rate 1 Gy/min; IBL-637, CIS-BioInternational, France). Cells were seeded in 6-well plates at a density of 5000 cells/well. To overcome the slow cell growth due to low seeding density, 30% “conditioned medium” was used. Conditioned medium was complete DMEM collected from flasks of BT20 cells and sterile filtered prior to use. Colonies were fixed with 4% v/v formaldehyde solution, stained with 0.4% w/v methylene blue and counted as described by Gill et al. [36]. Plating efficiencies were determined for each treatment condition and normalized to an untreated control to provide the survival fraction.

## Results

### TNBC cell lines with high and low MCT1 expression

**Fig. 1:**
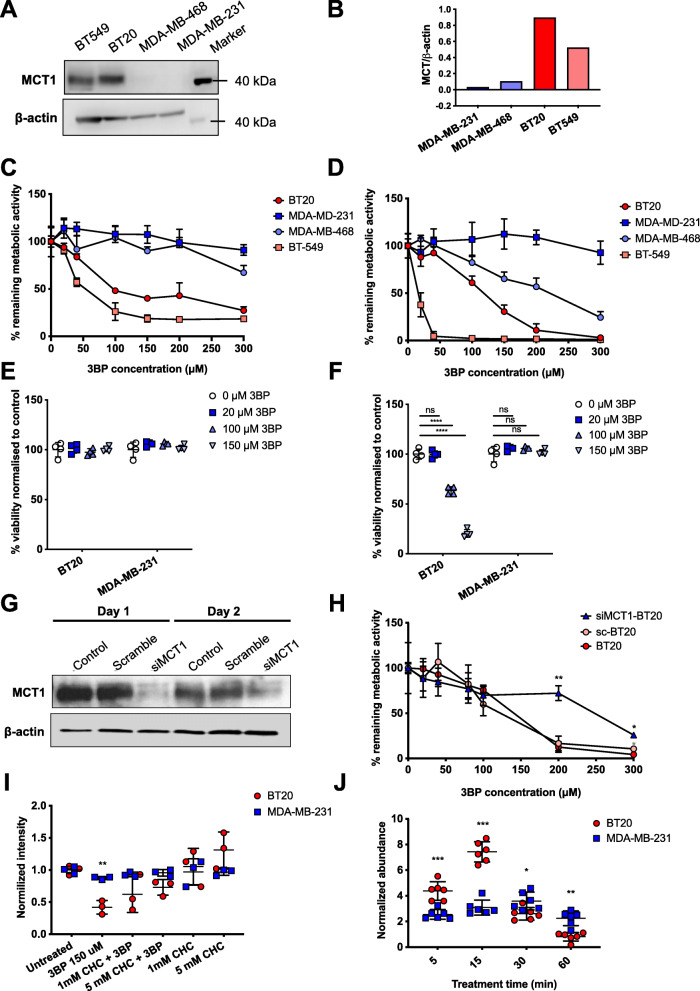
Differential cytotoxicity and uptake of 3-bromopyruvate (3BP) in breast cancer cells. (**A**) Western blot and (**B**) the relative quantification of the optical density of each band showing the expression of MCT1 in triple negative breast cancer (TNBC) cell lines MDA-MB-231, MDA-MB-468, BT20, and BT549. Remaining metabolic activity (%) at (**C**) 1 or (**D**) 24 h as a measure of cell viability. Cells were treated with a concentration range of 3BP (0-300 μM) and cell viability measured using the MTT assay (*n* = 4). Trypan blue exclusion after 3BP treatment (20, 100, or 150 μM) for (**E**) 1 or (**F**) 24 h was used as a metabolism-independent metric of cell viability for the measurement of the sensitivity of MDA-MB-231 and BT20 cells to 3BP. (**G**) Transfection efficiency was assessed by Western blot analysis 1 and 2 days after addition of 3BP. (**H**) BT20 cells transfected with scramble (control) siRNA (sc-BT20) or siRNA targeting MCT1 (siMCT1-BT20) were exposed to 3BP for 24 h and their viability measured (*n* = 4). (**I**) Cell viability following 24 h exposure of BT20 and MDA-MB-231 cells to α-cyano-4-hydroxycinnamate (CHC), an MCT1 inhibitor, and 3BP (150 μM). Data were normalized to untreated controls. (**J**) Bromide level (^78^Br and ^80^Br) normalized to control measured by LC-MS/MS in MDA-MB-231 and BT20 cell lysates (*n* = 6). Error bars represent SD, ** and * represent *P* < 0.05 and 0.01 respectively as determined by multiple *t* tests

Transcriptome-wide expression data from the Cancer Cell Line Encyclopedia (CCLE) were used to compare the expression of MCT1, MCT4, and MCT2 isoforms (encoded by *SLC16A1*, *3*, and *7* genes, respectively) in 57 breast cancer cell lines (Figure S2). BT20 and MDA-MB-231 were selected as representative MCT1-high/MCT4-low cells and MCT1-low/MCT4-high, respectively. According to CCLE data, both of these cell lines are also MCT2-low. Western blot analysis confirmed strong expression and undetectable expression of MCT1 (molar mass 54 kDa, apparent molar mass 40 kDa) in BT20 and MDA-MB-231 cells, respectively, while MCT4 was detected in MDA-MB-231 but not BT20 (Fig. 1a and b, Figure S1). In addition, BT549 (MCT1-high) and MDA-MB-468 (MCT1-low) TNBC cell lines were included in experiments to evaluate the dependence of the toxic effects of 3BP on MCT1. The expression of MCT1 in these cell lines is also shown in Fig. 1a and b.

### 3BP is selectively toxic to cells expressing MCT1

The effect of 3BP treatment on cell viability was evaluated using the MTT assay (Fig. 1c, d). 3BP was not toxic to MDA-MB-231 cells in concentrations up to 300 μM (> 90% cells still metabolically active). In contrast, 3BP caused a substantial decrease in the viability of BT20 cells, which express MCT1: a concentration of 100 μM reduced metabolic activity to 61% (*P* < 0.001) following a 24-h exposure (Fig. 1d). BT549 cells, also MCT1-expressing, were found to be completely metabolically inactive at the same concentration (Fig. 1d). Notably, this trend was already apparent after a short 1-h exposure of the same cell lines to 3BP (Fig. 1c), showing that the effects of 3BP occur rapidly, within 1 h. As 3BP interferes with cellular metabolism, cell viability was also assessed using a metabolism-independent dye (trypan blue) exclusion assay (Fig. 1e, f). It was hypothesized that although cells might be less metabolically active during short exposure times, they may retain viability. Indeed, it was found that cell membrane integrity was maintained (trypan blue excluded) for both BT20 and MDA-MB-231 cells following 1-h exposure, but that BT20 cells were selectively killed following a 24-h exposure.

To further evaluate the dependence of 3BP toxicity on MCT1 expression, two additional studies were carried out. First, siRNA-mediated silencing of MCT1 in BT20 cells was shown to confer resistance to 3BP treatment (Fig. 1g, h). For example, following treatment with 200 μM 3BP, the proportion of viable siMCT1-BT20 and wild type BT20 cells were 72% and 12%, respectively (*P* < 0.01). Treatment with 3BP started 24-h post-transfection and was terminated 48 h post-transfection, and importantly MCT1 expression was shown to remain low during this time (Fig. 1g). Second, both cell lines were co-treated with α-cyano-4-hydroxycinnamate (CHC), a validated MCT1 inhibitor. CHC did not reduce cell viability, but it partially prevented 3BP toxicity in BT20, with cell viability of 42% following 3BP alone but increasing to 62% and 73% when 3BP was combined with 1 mM or 5 mM CHC respectively (Fig. 1i). The addition of CHC to 3BP did not affect MDA-MB-231 cell viability.

### Intracellular bromide rapidly increases in MCT1-expressing cells following exposure to 3BP

According to the proposed mechanism of action of 3BP, bromide is the leaving group in a nucleophilic substitution reaction which results in a “pyruvylated” substrate and free bromide. Intracellular bromide (sum of ^78^Br and ^80^Br) in total cell lysates was used as a measure of cellular uptake of 3BP. Within 5 min of the addition of 3BP to the culture medium, bromide levels increased 4.38-fold and 2.55-fold in BT20 and MDA-MB-231 cells, respectively (1.8-fold difference, *P* < 0.001). The difference between the two cell lines increased further with time; by 15 min, FC was 7.42 and 3.08 for BT20 and MDA-MB-231 cells, respectively, *P* < 0.001 (Fig. 1j). For the later time points (up to 60 min), the intracellular content of bromide decreased in BT20 cells, while in MDA-MB-231 cells, bromide content did not change significantly. Results for intracellular bromide levels for sc-BT20 and siMCT1-BT20 are shown in Figure S3.

### 3BP inhibits GAPDH but not hexokinase

**Fig. 2:**
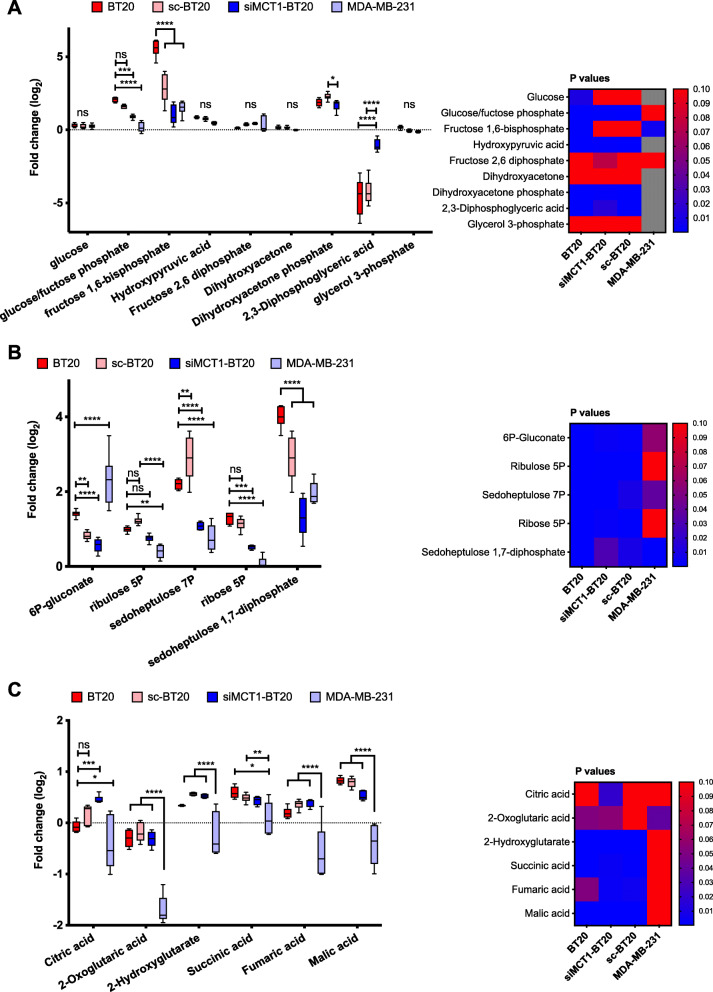
Effects of 3-bromopyruvate (3BP) on glucose metabolism. Changes in (**A**) glycolysis, (**B**) pentose phosphate pathway (PPP), and (**C**) tricarboxylic acid (TCA) cycle in response to 1-h treatment with 100 μM 3BP (*n* = 6). Please refer to Fig. 3 for a summary of these pathways. Data are expressed as fold change (log_2_(FC)) compared to untreated controls for each cell line. The heatmaps show the statistical significance (*P* value < 0.05) of each metabolite change within each cell line. Gray squares in the heatmap indicate missing data. The error bars represent the standard deviation from the mean. *P < 0.05, **P < 0.01, ***P < 0.001. Asterisks, statistical significance of the difference between BT20 cells and each other cell type, unless otherwise shown. Statistical significance was calculated using two-way ANOVA, corrected for multiple comparisons by the Tukey test. The multiplicity adjusted *P* value is presented

**Fig. 3:**
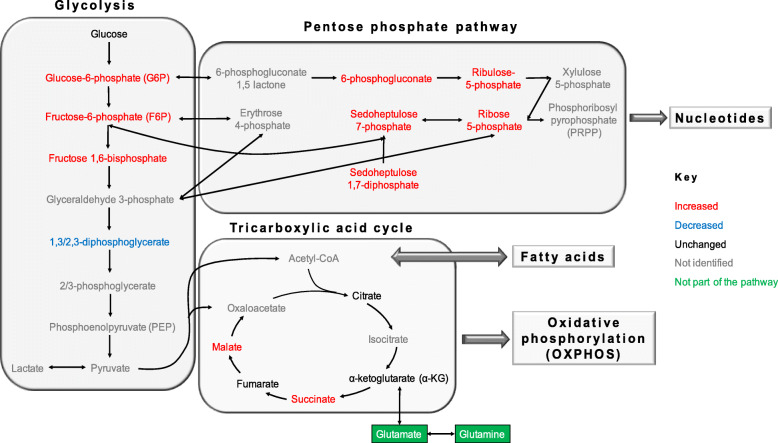
Diagram of the overall changes in glycolysis, TCA, and PPP in BT20 cells following exposure to 3BP (enzymes and co-factors not included). Intermediates that were increased, decreased, unchanged, or not identified are annotated in red, blue, black, and gray respectively. Related metabolites or processes that are not part of the presented pathways are shown in green

Changes in the metabolites involved in the metabolism of glucose, specifically, glycolysis, the pentose phosphate pathway (PPP) and the tricarboxylic acid cycle (TCA) are presented in Fig. 2 and qualitatively summarized in Fig. 3. For each metabolite, the fold-change (FC) of each cell sample is presented as the boxplot per pathway. The statistical comparisons in the boxplots refer to the differences between the groups, highlighting the dependence of the change on MCT1 expression. The accompanying heat maps show the *P* values of the metabolite change within each cell line, with statistically significant changes (*P* < 0.05) in blue. The addition of 3BP resulted in a marked accumulation of the intermediates that follow the HK reaction (glucose/fructose phosphates) in BT20 but not in MDA-MB-231 or siMCT1-BT20 cells (BT20 vs siMCT1-BT20 or MDA-MB-231, *P* < 0.001) (Fig. 2a). Conversely, 1,3/2,3-biphosphoglycerate, the intermediate immediately following the GAPDH reaction, was selectively depleted in MCT1-positive cells, *P* < 0.0001 (Fig. 2a). Together, these observations are consistent with GAPDH but not HK inhibition by 3BP.

### Pentose phosphate pathway is upregulated in response to 3BP

Given the inhibition of the glycolytic GAPDH, the proportion of glucose/fructose-6P entering the pentose phosphate pathway (PPP) might be expected to increase (Fig. 3). Accordingly, the results presented in Fig. 2b indicate differential upregulation of PPP metabolites in MCT1-expressing cells (with the exception of 6P-gluconate), both in the oxidative (ribulose-5P, ribose-5P) and the non-oxidative branch (sedoheptulose phosphates). Upregulation of the PPP is a cellular response to oxidative stress and/or need for nucleic acid precursors.

### The TCA metabolite abundances are not significantly altered by 3BP treatment

The levels of all detected TCA cycle metabolites remained close to the baseline level (log_2_(FC)<1). Relatively small differences between the different cell types were observed. These changes in MCT1-expressing BT20 cells are summarized qualitatively in Fig. 3. Tight distributions were found in BT20, sc-BT20 and siMCT1-BT20 cell lysates but very broadly distributed levels for all metabolites were observed for MDA-MB-231 cell lysates. A marked decrease in 3-oxo-glutarate was observed in 3BP-treated MDA-MB-231 cells, compared to baseline. Succinate is the only intermediate that showed a slight but significant accumulation in BT20 and sc-BT20 cells compared to MDA-MB-231 cells (Fig. 2c). Dehydrogenation of succinate to fumarate is catalyzed by succinate dehydrogenase, which is inhibited by malonate an intermediate that is also upregulated in BT20 cells (log_2_(FC) = 0.77 and 0.33 for BT20 and siMCT1-BT20, respectively, *P* < 0.001). Note that although malonate is not shown in Fig. 2, it was identified in the metabolomics analysis. Crucially, TCA can be fuelled by glutaminolysis via α-ketoglutarate (αKG). All treatments were carried out in complete growth medium, where glutamine is abundant.

### Glutathione and NADH are depleted at early time points

**Fig. 4:**
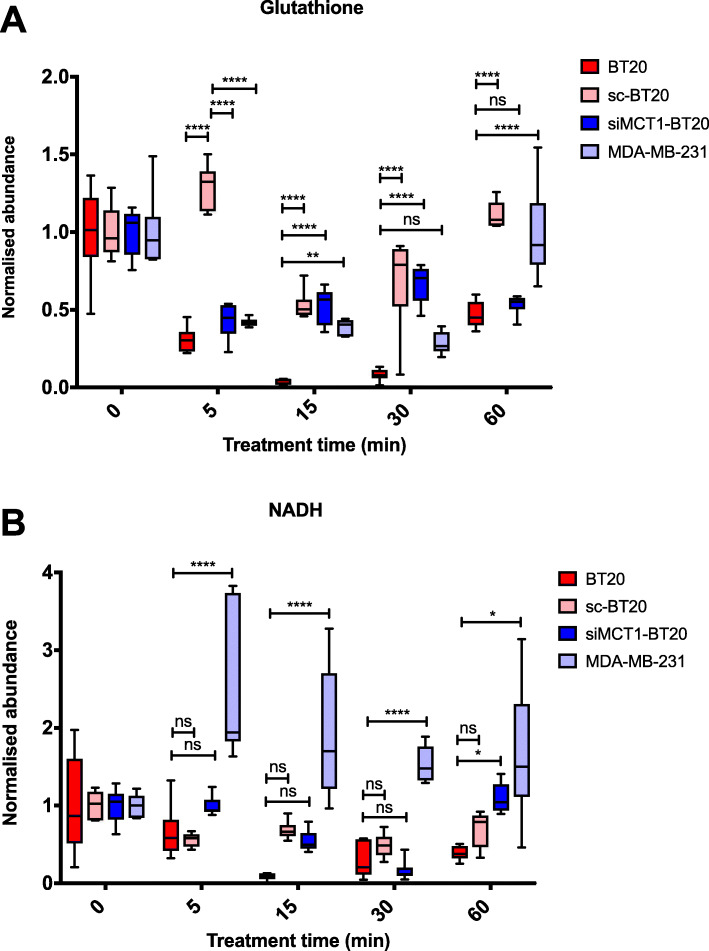
Changes in the levels of glutathione and nicotinamide dinucleotide. Changes in (**A**) glutathione and (**B**) nicotinamide dinucleotide (NADH) over time following addition of 100 μM 3BP. Measurements from independent samples were taken for each time point (*n* = 6). Metabolite levels are presented as abundance normalized to untreated control for each cell type and represent intracellular levels. The error bars represent the standard deviation from the mean. *P < 0.05, **P < 0.01, ***P < 0.001. Statistical significance was calculated using two-way ANOVA, corrected for multiple comparisons by the Tukey test. The multiplicity adjusted *P* value is presented

Evidence that MCT1-expressing cells undergo oxidative stress when treated with 3BP was provided by the upregulation of PPP (Fig. 2b), a major pathway in cellular defense against oxidative stress. PPP is the main cytosolic source of NADPH. Although NADPH was not directly detected, the upregulation of ribose-5P, the product of the first non-oxidative reaction, confirms the oxidative three-step conversion of glucose-6P to ribulose-5P (oxidative strand of PPP), is operating, thus producing NADPH (2 eq). Further, GSH and NADH were both simultaneously depleted (Fig. 4), with lowest levels within 15-30 min of initial exposure to 3BP. This was different from the metabolic pathways presented in Fig. 2, in which the most significant changes were observed at 1 h (data not shown for 5, 15, and 30 min). Sc-BT20 cells presented anomalous behavior with regard to glutathione, with a pattern more closely resembling that of siMCT1-BT20 cells than the wild type. This suggests that this behavior could be related to the transfection with siRNA. Partial recovery was observed by 60 min. In contrast to NADPH, GSH can be “pyruvylated” by 3BP hence its regeneration in the presence of NADPH is not possible. Glycolysis is a cytosolic source of NADH, crucially at the GAPDH step, where NAD^+^ is reduced to NADH, which is in agreement with the observed depletion of NADH.

### Nucleotide synthesis is downregulated after 3BP treatment

**Fig. 5:**
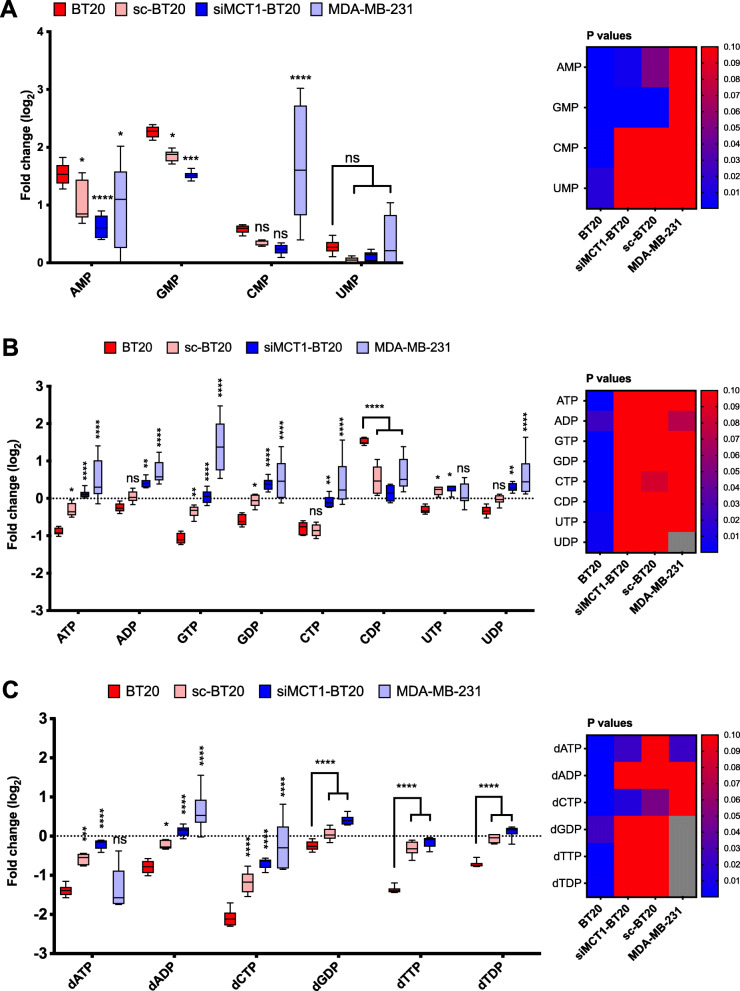
Levels of nucleotides after treatment with 3BP. The level of nucleotides following treatment with 3BP (100 μM) for 1 h (*n* = 6): (**A**) monophosphates, (**B**) di- and triphosphates, and (**C**) deoxyribonucleotides. The heatmaps show the statistical significance (*P* value < 0.05) of each metabolite change within each cell line. Gray squares in the heatmap indicate missing data. Error bars represent the standard deviation from the mean. *P < 0.05, **P < 0.01, ***P < 0.001. Asterisks indicate statistical significance of the difference between BT20 cells and each other cell type, unless otherwise shown. Statistical significance was calculated using two-way ANOVA, corrected for multiple comparisons by the Tukey test. The multiplicity adjusted *P* value is presented

The level of nucleotides deviated furthest from baseline in BT20 compared to other cells following exposure to 3BP (Fig. 5). The mean level of ATP was lowest in BT20 cells (log_2_(FC) = −0.89) (Fig. 5b), consistent with the observed inhibition of glycolysis in this cell line on exposure to 3BP. MDA-MB-231 and siMCT1-BT20 cells did not show an overall decrease in ATP: log_2_(FC) = 0.49 and 0.12, respectively. Further, the three nucleotides whose levels were found to be increased were monophosphates (AMP, GMP, and CMP) (Fig. 5a) while di- and tri-phosphates were decreased in BT20 cells (apart from CDP) (Fig. 5b). Also, there is a difference between deoxyribonucleotides (Fig. 5c). For example, dCTP was significantly depleted in BT20 and sc-BT20 cells (log_2_(FC) = −2.09 and −1.18, respectively) but not as markedly in siMCT1-BT20 and MDA-MB-231 cells (log_2_(FC) = −0.69 and −0.23, respectively), although values were statistically significant for all groups (*P* < 0.0001). Declining ATP levels were consistent with the lower levels of di- and tri-phosphates despite the increase in ribose-5P levels, which is an essential substrate for nucleotide synthesis.

### 3BP selectively decreases the acidification of culture medium

**Fig. 6:**
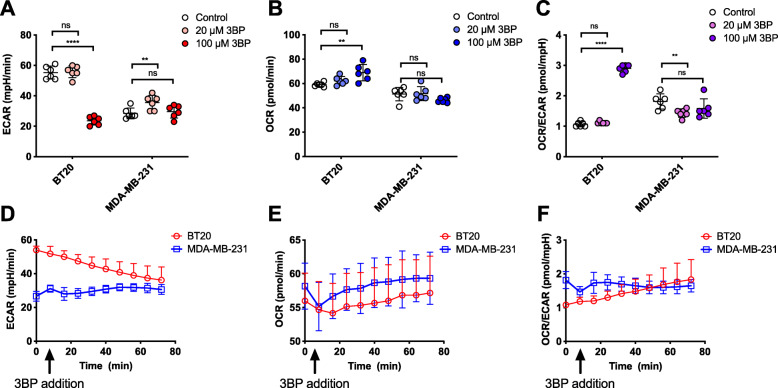
Effect of 3BP on the extracellular acidification rate (ECAR), oxygen consumption rate (OCR), and the OCR/ECAR ratio of TNBC cells. BT20 and MDA-MB-231 cells were treated with 20 or 100 μM 3BP for 1 h in DMEM prior to measurement of (**A**) EACR, (**B**) OCR, and (C) OCR/EACR. For the time-course measurements of (**D**) EACR, (**E**) OCR, and (**F**) OCR/EACR, 3BP was injected through the cartridge ports for a final concentration of 100 μM in the well, following a baseline measurement for each group. Statistical significance was calculated using “unmatched” two-way ANOVA, with the *P* value corrected for multiple comparisons using the Sidak test. Multiplicity adjusted *P* value is reported. *N* = 4. ns *P* > 0.05, *P < 0.05. Error bars represent the standard deviation from the mean

Measurements of extracellular media acidification rate (ECAR) and oxygen consumption rate (OCR) using a Seahorse XF96 analyzer showed that ECAR, a metric for glycolytic metabolism, was selectively decreased in BT20 cells (55.3 to 23.7 mpH/min, *P* < 0.0001) but not in MDA-MB-231 cells upon exposure to 100 μM 3BP for 1 h (Fig. 6a). Simultaneously, a less dramatic but statistically significant increase in the OCR signifies that 3BP causes a shift to oxidative metabolism selectively in BT20 cells (*P* < 0.01) (Fig. 6b). Combined, these effects result in a rapid and substantial shift toward oxidative metabolism, evidenced by the OCR/ECAR ratio (Fig. 6c). To further elucidate the kinetics of this shift, 3BP was injected via the cartridge ports following a baseline measurement and the change in ECAR, OCR, and OCR/ECAR was recorded in real-time (Fig. 6d, e, and f). The smaller observed effect demonstrated by the time-course experiment is expected as in this case the measurements occur in real-time while there is a significant time interval between cell treatment and measurement when pre-treated cells are used. Although a sub-toxic concentration of 3BP (20 μM) did not result in statistically significant changes within 1 h (Fig. 6a, b, and c), prolonged incubation with 20 μM 3BP for 24 h resulted in a statistically significant selective decrease in ECAR (Figure S4A, 69 to 55.5 mpH/min, *P* < 0.05), although at this low concentration, glycolytic inhibition, as indicated by the low ECAR, was not accompanied by a significant increase in the OCR (Figure S4B), indicating unaltered oxidative metabolism.

### 3BP reduces clonogenic survival of MCT1-expressing cells, potentiating external beam radiation

**Fig. 7:**
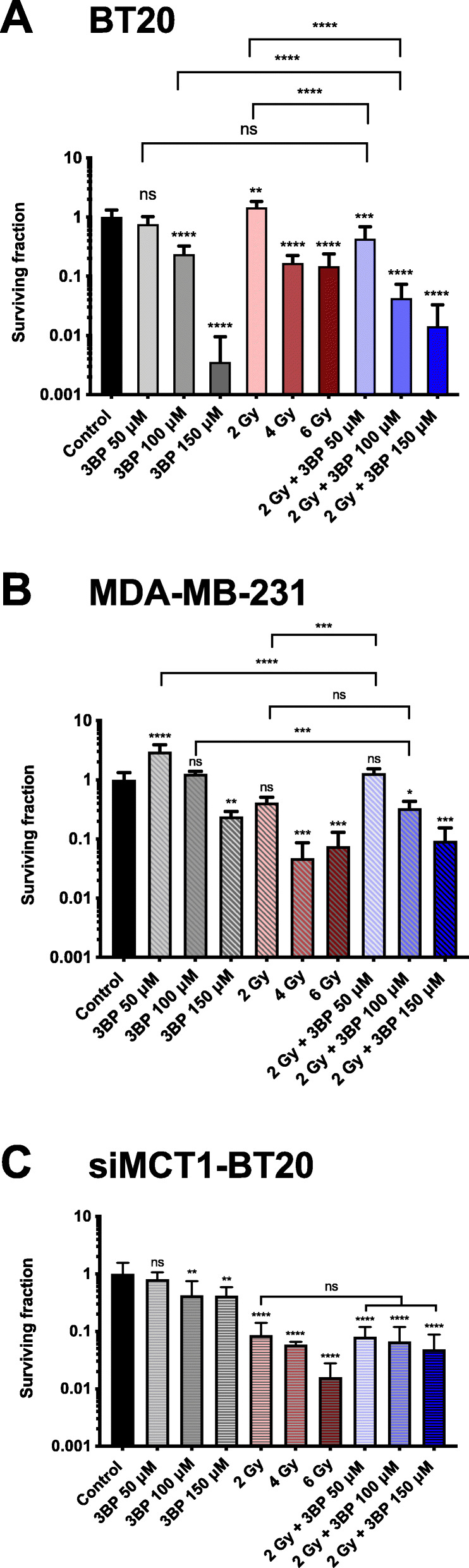
Colony formation assay showing the effects of 3-bromopyruvate (3BP) and ionizing radiation on triple-negative breast cancer cells. (**A**) MCT1-positive BT20 cells, (**B**) MCT1-negative MDA-MB-231 cells, and (**C**) MCT1-knockdown BT20 cells (siMCT1-BT20) were treated with a 50, 100, or 150 μM of 3BP for 1 h, a range of doses of ionizing radiation, or a combination of both where irradiation followed the 3BP treatment. siMCT1-BT20 cells were treated with 3BP and/or radiation and seeded at clonogenic density within 48-h post-transfection. For all groups, *n* = 6. Statistical significance was assessed with unmatched one-way ANOVA, corrected for multiple comparisons by the Tukey test. Multiplicity adjusted *P* value is reported. ns *P* > 0.05, *P < 0.05, **P < 0.01, ***P < 0.001, ****P < 0.0001. Unless otherwise marked, star symbols refer to comparisons of each treatment group with the control. Error bars show the standard deviation from the mean

The impact of ionizing radiation on cell survival, alone, or in combination with 3BP pre-treatment, was evaluated using clonogenic assays. Treatment with 50 μM 3BP had minimal impact on clonogenic survival on BT20 cells, in agreement with the MTT assay (Fig. 7a). In contrast, 100 and 150 μM 3BP substantially decreased cell survival (surviving fraction, SF 0.23 and 0.003, respectively, *P* < 0.0001). The lowest radiation dose used (2 Gy) was not cytotoxic in BT20 cells, while 4 and 6 Gy resulted in marked reduction in the number of colonies (SF 0.17 and 0.15, respectively, *P* < 0.0001 compared to control). The combination of 50 μM 3BP with 2 Gy did not decrease the SF significantly compared to 50 μM 3BP alone. However, an additive contribution of a sub-toxic radiation dose (2 Gy) to the 3BP-induced cytotoxicity was observed with 100 μM 3BP (SF 0.04 for combination compared to SF 0.23 for 3BP alone, *P* < 0.0001). In contrast, potentiation of the efficacy of ionizing radiation was not observed for either of the MCT1-negative controls (MDA-MB-231 and siMCT1-BT20) (Fig. 7b, c). The siMCT1-BT20 cells were strikingly more sensitive to radiation alone compared to the wild type BT20 cell line. For example, 2 Gy decreased the SF of siMCT1-BT20 to 0.08 compared to having no effect on BT20 (*P* < 0.001). Notably, both MDA-MB-231 exposed to 150 μM 3BP and siMCT1-BT20 exposed to 100 and 150 μM 3BP did show a low level of response to 3BP although its effect on viability was markedly less than that observed in BT20 cells. This may reflect the influx of low levels of 3BP by other routes or, in the case of siMCT1-BT20, incomplete knockdown of MCT1 as would be expected with the use of siRNA.

## Discussion

TNBC is an aggressive form of breast cancer with poor outcomes compared to hormone or anti-ERBB2 therapy responsive groups, partly because patients with TNBC do not benefit from treatments that target the hormone receptors or ERBB2. The treatment landscape for patients with TNBC remained largely static until the recent approval of PARP inhibitors and atezolizumab, an antibody against anti-PD-L1 (programmed cell death ligand 1), in combination with chemotherapy [5, 37, 38]. Although promising, only a subset of patients benefits from these targeted treatments and new treatment options are urgently needed.

The Warburg phenotype in breast cancer has been associated with basal-like, MYC-driven, hormone receptor negative cell lines and tumors [7, 39, 40]. These tumors are likely to rely on MCTs to export lactate to sustain their glycolytic metabolism, and MCT1 expression is correlated with basal-like TNBC phenotype [16, 17]. Thus, MCT1 is an attractive target for glycolytic tumors, including TNBC. Interest in targeting the MCTs, in particular MCT1, has increased in the last decade, leading to the development of two chemically distinct classes of specific MCT1/2 inhibitors (MCT1/2i) [22, 23, 41]. Although inhibitors efficiently block lactate transport through the transporter, results to date indicate that expression of MCT1 may not be sufficient for cytotoxicity in vitro and in vivo. Various mechanisms can confer resistance to MCT1i treatment such as the co-expression of other MCT isoforms or a switch to oxidative metabolism. For example, Morais-Santos et al. have demonstrated that siRNA-mediated MCT1 knockdown reduced tumor growth of BT20 cells but MDA-MB-468 tumors continued to grow even with simultaneous MCT1 and MCT4 knockdown [42]. For breast cancer cell lines, MCT4 is frequently co-expressed with MCT1, as revealed by comparing expression data available on the CCLE database (Figure S2). In this study, we focus on an alternative strategy, originally proposed by Ko and co-workers [28], which exploits the high expression of MCT1 to deliver a toxic molecule, 3BP, to cells expressing the transporter. Birsoy et al. [26] showed that MCT1 expression is sufficient to sensitize cancer cells to 3BP. Our results support these findings showing the impact of 3BP on cell viability is higher for MCT1-expressing versus non-expressing cells. Furthermore, we show, in agreement with Morais-Santos et al. that CHC, an MCT1 inhibitor, at concentrations up to 5 mM, is not toxic to MCT1-positive breast cancer cells, such as BT20. MCT4 is unlikely to provide resistance to 3BP treatment because 3BP reacts with available thiols once in the cell [27]. Another TNBC cell line, BT549, which expresses both MCT1 and 4, was found to be sensitive to 3BP treatment (almost 100% reduction in metabolic activity following a 24-h exposure to 100 μM 3BP, Fig. 1d).

Lactate contributes to the hostility of the tumor microenvironment by lowering the pH, enhancing immune escape, promoting angiogenesis and facilitating invasion and metastasis [20]. In this study, a short (1 h) exposure to 3BP simultaneously lowered the ECAR (a surrogate for glycolytic metabolism) and elevated the OCR (indicative of oxidative metabolism) in BT20 but not MDA-MB-231 cells, indicating that 3BP decreases lactate efflux selectively in MCT1-expressing cells. This reflects the effects of small molecule MCT1 inhibitors which have also been found to decrease ECAR [41]. We speculate that this is independent of MCT4 co-expression since it is caused by direct inhibition of glycolysis, rather than inhibition of lactate export. Upregulation of glycolysis confers resistance to apoptosis, as well as resistance to chemo- and radiotherapy. Motivated by studies showing potentiation of the cytotoxic effects of radiotherapy by both MCT1 inhibitors [43, 44] and anti-glycoltytic compounds [10, 45], we tested the combination of 3BP plus a single dose of ionizing radiation (2 Gy). The results provide evidence that a short (1 h) treatment with 100 μM 3BP, shown to cause ~40% reduction in metabolic activity (Fig. 1), combined with a sub-toxic radiation dose was more effective in reducing the clonogenic survival of TNBC cells than 3BP alone. This constitutes a promising outcome, which replicates the radio-potentiating effects of both MCT1 inhibitors [43, 44] and glucose analogs [10], while remaining selective and unlikely to be affected by MCT4 expression. Importantly, this radio-potentiating effect was not seen in MCT1-knockdown BT20 cells or MDA-MB-231 cells. In accordance with previous published reports of the radiosensitizing effect of MCT1 inhibition, siMCT1-BT20 cells were found to be dramatically more sensitive to radiation than their MCT1-expressing counterparts (Fig. 7c) or MDA-MB-231 cells, which have naturally low MCT1 expression (Fig. 7b). Aspects of the treatment such as radiation and 3BP dose, treatment time and interval between the two treatments, as well as combination, for example, with a PPP inhibitor will be explored in future studies.

The alteration in metabolism caused by 3BP in breast cancer cells expressing MCT1 may be therapeutically exploitable. The observed pattern, accumulation of early glycolytic intermediates with concomitant depletion of late intermediates, is consistent with the reduction in ECAR, and denotes inhibition of the glycolytic pathway. Specifically, these results indicate that GAPDH, but not hexokinase 2 (HK-2), is inhibited. GAPDH has a thiol (-SH) active center and has been shown to be inhibited not only by 3BP but also by similar compounds such as iodoacetate [46, 47]. The observation that NADH is depleted following 3BP also supports the notion that GAPDH is inhibited. The later partial recovery of NADH can be attributed to glutaminolysis. Inhibition of both GAPDH and HK-2 by 3BP has been shown in several studies; however, a recent study by Jardim-Messeder et al. [48] demonstrated that a 1-h treatment with 100 μM 3BP did not suppress HK-2 activity in hepatocellular carcinoma cells (HepG2), as the data presented here also suggest. Partial inhibition of HK-2 cannot be excluded through metabolomics, since accumulation of glucose/fructose phosphates can result from inhibition downstream from the HK-2 reaction.

TCA and PPP are tightly coordinated with glycolysis and were, therefore, also investigated. In line with GAPDH inhibition and retention of HK-2 function, the upregulation of PPP intermediates was observed. This reflects a cellular response to oxidative stress and driven by the need for nucleotide synthesis for proliferation and/or DNA repair. The pro-oxidant activity of 3BP is in agreement with previous studies showing that it reacts directly with GSH with the generation of reactive oxygen species (ROS) [12, 49, 50]. The induction of oxidative stress, revealed by the upregulation of PPP and depletion of GSH, is a mechanism of action of 3BP that is linked to but distinct from inhibition of glycolysis. Apart from directly reacting with 3BP, it is also possible that GSH acts as a scavenger for reactive oxygen species (ROS) generated by 3BP [27, 49, 51]. Combined with a PPP inhibitor, the pro-oxidant effects of 3BP could be potentiated and this can be considered for future studies to synergize with radiotherapy.

Nucleotides are the immediate precursors for the synthesis of nucleic acids, and are, therefore essential for proliferation and DNA repair in response of ionizing radiation. Intriguingly, although the level of ribose-5P, an essential precursor for nucleotide synthesis, was elevated, most nucleotides, including ATP, were depleted. Phosphorylation of ATP occurs in the mitochondria and its depletion, a consequence of glycolytic inhibition, could be responsible for the downregulation of nucleotide synthesis. Although there are reports showing interference of 3BP with mitochondrial metabolism [48, 52, 53], the abundance of TCA cycle metabolites was not found to be markedly affected in this study, with the unexpected exception of 2-oxo-glutarate (or, α-ketoglutarate, αKG) in MDA-MB-231 cells. The cause of this effect is unclear but it is possible that the function of other monocarboxylate transporters could be involved. Although out of the scope of this study, the potential relevance of the mitochondrial pyruvate carrier (MPC), which is responsible for the capture of pyruvate into the mitochondrion, could be explored. The explanation for the generally unperturbed function of the TCA cycle could be that other pathways can replenish TCA intermediates through anaplerosis. Importantly, the growth medium used in the experiments reported here contained glutamine (0.29 mg/mL) which can directly fuel the TCA via αKG. As Jardim-Messeder et al. showed using HepG2 cells, glutaminolysis is not directly inhibited by 3BP (100 μM) and it can continue to fuel the TCA cycle [48]. Glutaminolysis also generates NADH, which could be responsible for the observed partial recovery of NADH at 60 min (Fig. 3).

## Conclusions

Overall, this study adds to a body of evidence showing that 3BP is a potent and promising therapeutic agent for tumors expressing MCT1. Using cell lines of the same tumor type, as well as controls with silenced MCT1, we provide evidence that the cytotoxic effects of 3BP are dependent on MCT1 expression. Using an unbiased, comprehensive metabolomics analysis, the simultaneous effects of 3BP on a number of metabolic processes was investigated. 3BP induces metabolic perturbation which impacts several crucial aspects of cancer metabolism—glycolysis, redox balance, and nucleotide synthesis—suggesting that cells are unlikely to be able to acquire resistance to 3BP. It was also demonstrated that combining 3BP with ionizing radiation, a treatment that is frequently used for TNBC, potentiates its cytotoxicity. Expression of MCT1, low tumor pH, and upregulated glycolysis are all features uniquely exploited by 3BP for its selective uptake and toxicity. Safety concerns reasonably arise about the clinical translation of this compound. Despite its remarkable local selectivity, it can react with proteins and peptides in circulation and in healthy tissues. Therefore, targeting 3BP at the tumor site is essential to harness the full potential of this promising compound. This can be achieved by a targeted delivery strategy, which is currently under development.

## Supplementary Information

**Additional file 1: Supplementary methods**. **Table S1.** Charge, mass-to-charge ratio (m/z), retention time and formula of identified metabolites. **Figure S1.** Full western blots. (A) MCT1 expression in triple negative breast cancer (TNBC) cell lines, as presented in Fig. 1a. (B) MCT1 expression in native BT20 cells and BT20 cells transfected with siRNA against MCT1 (siMCT1), 1 and 2 days post-transfection, as presented in Fig. 1g. (C) Western blots for the assessment of MCT1 and MCT4 expression in the TNBC cell lines, BT20 and MDA-MB-231. **Figure S2.** RNA expression data for breast cancer cell lines. RNA expression of isoforms MCT1, 2 and 4, encoded by SLC16A1, 7 and 3, respectively were plotted for breast cancer cell lines. The RNA expression data for each isoform were derived from the Cancer Cell Line Encyclopedia (CCLE) database, expressed as log_2_(Fold Change) and plotted using Prism (GraphPad, CA, USA). **Figure S3.** Bromide level (^78^Br and ^80^Br) normalized to control measured by LC-MS/MS in lysates from BT20 cells and BT20 cells transfected with siRNA targeted against MCT1 (siMCT1-BT20) or scramble siRNA (sc-BT20) (n=6). 3BP uptake is significantly different between BT20 and siMCT1-BT20 at all time points, but no differences evident when comparing BT20 to the scrambled control cells. Error bars represent SD, **** represents *P*<0.001 as determined by multiple t-tests. **Figure S4.** Effect of sub-toxic concentration of 3BP on the extracellular acidification rate (ECAR), oxygen consumption rate (OCR) and the OCR/ECAR ratio of TNBC cells. BT20 and MDA-MB-231 cells were treated with 20 μM 3BP for 24 h in DMEM prior to measurement of the OCR and ECAR. Statistical significance was calculated using ‘unmatched’ two-way ANOVA, with the *P* value corrected for multiple comparisons using the Sidak test. Multiplicity adjusted *P* value is reported. *N*=4. Ns *P*>0.05, *P<0.05. Error bars represent the standard deviation from the mean.

## Data Availability

The datasets used and/or analyzed during the current study are available from the corresponding author on reasonable request.
