# Supplementary material for: 3-Bromopyruvate-mediated MCT1-dependent metabolic perturbation sensitizes triple negative breast cancer cells to ionizing radiation
Source: Cancer Metab. 2021 Oct 14;9:37. doi: 10.1186/s40170-021-00273-6 (PMC8515664; doi:10.1186/s40170-021-00273-6)
Supplement: Supplementary file 1 — Additional file 1: Supplementary methods. Table S1. Charge, mass-to-charge ratio (m/z), retention time and formula of identified metabolites. Figure S1. Full western blots. (A) MCT1 expression in triple negative breast cancer (TNBC) cell lines, as presented in Fig. 1a. (B) MCT1 expression in native BT20 cells and BT20 cells transfected with siRNA against MCT1 (siMCT1), 1 and 2 days post-transfection, as presented in Fig. 1g. (C) Western blots for the assessment of MCT1 and MCT4 expression in the TNBC cell lines, BT20 and MDA-MB-231. Figure S2. RNA expression data for breast cancer cell lines. RNA expression of isoforms MCT1, 2 and 4, encoded by SLC16A1, 7 and 3, respectively were plotted for breast cancer cell lines. The RNA expression data for each isoform were derived from the Cancer Cell Line Encyclopedia (CCLE) database, expressed as log2(Fold Change) and plotted using Prism (GraphPad, CA, USA). Figure S3. Bromide level (78Br and 80Br) normalized to control measured by LC-MS/MS in lysates from BT20 cells and BT20 cells transfected with siRNA targeted against MCT1 (siMCT1-BT20) or scramble siRNA (sc-BT20) (n=6). 3BP uptake is significantly different between BT20 and siMCT1-BT20 at all time points, but no differences evident when comparing BT20 to the scrambled control cells. Error bars represent SD, **** represents P<0.001 as determined by multiple t-tests. Figure S4. Effect of sub-toxic concentration of 3BP on the extracellular acidification rate (ECAR), oxygen consumption rate (OCR) and the OCR/ECAR ratio of TNBC cells. BT20 and MDA-MB-231 cells were treated with 20 μM 3BP for 24 h in DMEM prior to measurement of the OCR and ECAR. Statistical significance was calculated using ‘unmatched’ two-way ANOVA, with the P value corrected for multiple comparisons using the Sidak test. Multiplicity adjusted P value is reported. N=4. Ns P>0.05, *P<0.05. Error bars represent the standard deviation from the mean. [file 40170_2021_273_MOESM1_ESM.docx]

## SUPPLEMENTARY INFORMATION

# **3-Bromopyruvate-Mediated MCT1-Dependent Metabolic Perturbation Sensitizes Triple Negative Breast Cancer Cells to Ionizing Radiation**

### **SUPPLEMENTARY METHODS**

#### *Metabolomics*

Method 1: IC-MS/MS

Anion-exchange chromatography was performed using an ICS-5000+ HPLC system incorporating an electrolytic anion generator (KOH) which was programmed to produce a OH^–^ gradient over 37 min. An inline electrolytic suppressor removed OH^–^ ions and cations from the post-column eluent stream prior to MS analysis (Thermo Scientific Dionex AERS 500). A 10 μL partial loop injection was used for all analyses and the chromatographic separation was performed using a Thermo Scientific Dionex IonPac AS11-HC 2 × 250 mm, 4 μm particle size column. The IC flow rate was 0.250 mL/min. The total run time was 37 min and the hydroxide ion gradient comprised as follows: 0 mins, 0mM; 1 min, 0 mM; 15 min, 60 mM; 25 min, 100 mM; 30 min, 100 mM; 30.1 min, 0 mM; 37 min, 0 mM. Analysis was performed on a Thermo Scientific Q Exactive equipped with a HESI II probe, in negative ion mode using a scan-range from *m/z* 60-900 and resolution set to 70,000. The tune file source parameters were set as follows: Sheath gas flow 60 mL/min; Aux gas flow 20 mL/min; Spray voltage 3.6 V; Capillary temperature 320^o^C; S-lens RF value 70; Heater temperature 350^o^C. MS scan parameters were set as follows: Automatic gain control target was set to 1e6v ions and the Max injection time value was 250 ms. The column temperature was kept at 30°C throughout the experiment. Full scan data were acquired in continuum mode.

*Method 2: C18 Reversed Phase (underivatized samples)*

C18 reversed-phase analysis of underivatized samples was performed using a Thermo UltiMate 3000 UHPLC system with a gradient elution program coupled directly to a Thermo Scientific Q Exactive mass spectrometer equipped with a HESI II probe. A 5 μL partial loop injection was used for all analyses with pre- and post-injection wash program. A Waters CORTECS UPLC T3 1.6µm (2.1x100mm) column was used with a flow rate of 0.40 mL/min. The total run time was 18 mins. Mobile phase A comprised milli-Q water with 0.1% formic acid and mobile phase B was methanol with 0.1% formic acid. The gradient elution program was as follows: 0 mins, 5% B; 4 min, 50% B; 12 min, 99% B; 15 mins, 99% B; 15.1 min, 5% B; 18 min, 5% B. The column temperature was kept at 40°C throughout the experiment. Analysis was performed in positive and negative ion mode separately using a scan-range from *m/z* 60-900 and resolution set to 70,000. The tune file source parameters were set as follows: Sheath gas flow 60 mL/min; Aux gas flow 20 mL/min; Spray voltage 3.6v; Capillary temperature 320^o^C; S-lens RF value 70; Heater temperature 350^o^C. MS scan parameters were set at follows: Automatic gain control target 5e6 ions and the Max injection time value was 120ms. Full scan data were acquired in continuum mode. A data directed tandem mass spectrometry method was utilized (ddMS2) with no inclusion list. The scan parameters for ddMS2 were as follows: Microscans 2, resolution 17,500, AGC target 5e4 ions, maximum IT 80ms, loop count 10 and NCE 35.

Method 3: C18 Reversed phase (derivatized samples)

The third LC-MS method used a sample derivatisation protocol followed by analysis based on a modified version of the Waters AccQ-Tag method (Salazar et al., 2011). C18 reversed-phase analysis of derivatized samples was also performed using a Thermo UltiMate 3000 UHPLC system coupled directly to a Thermo Scientific Q Exactive mass spectrometer equipped with a HESI II probe. A 5 μL partial loop injection was used for all analyses with pre- and post-injection wash program. A Waters AccQ-Tag column (2.1x100 mm) was used with a flow rate of 0.50 mL/min. The total run time was 9.5 min. Mobile phase A and B comprised commercially available AccQ-Tag reagents prepared as recommended by Waters (Waters PLC, Elstree, UK). The gradient elution program was modified from the published AccQ-Tag method as follows: 0 min, 0.1%B; 0.54 min, 9.1%B; 5.74 min, 21.2%B; 7.74 min, 59.6%B; 8.04 min, 90%B; 8.05 min, 90%B; 8.64 min, 0%B; 9.5 min, 0.1% B. The column temperature was kept at 40°C throughout the experiment. Analysis was performed in positive ion mode separately using a scan-range from *m/z* 70-1050 and resolution set to 70,000. The tune file source parameters were set as follows: Sheath gas flow 60 mL/min; Aux gas flow 20 mL/min; Spray voltage 3.6 V; Capillary temperature 320^o^C; S-lens RF value 70; Heater temperature 350^o^C. MS scan parameters were set as follows: Automatic gain control target 3e6 ions and the Max injection time value was 200 ms. Full scan data were acquired in continuum mode.

**Table S1:** Charge, mass-to-charge ratio (m/z), retention time and formula of identified metabolites


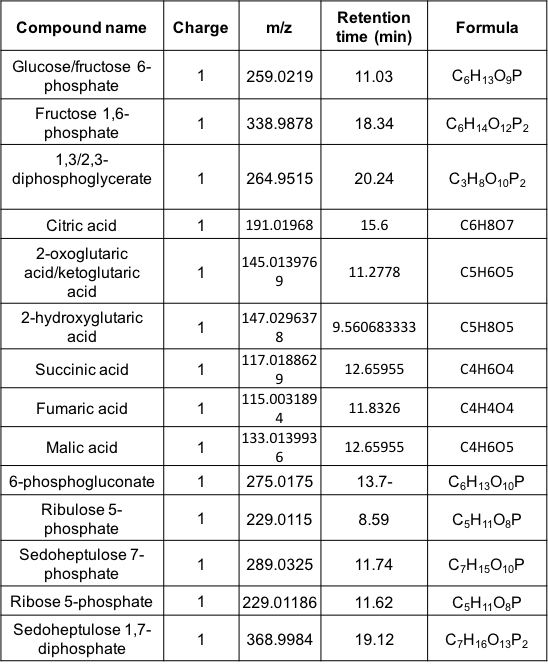


**Figure S1: Full western blots**. (A) MCT1 expression in triple negative breast cancer (TNBC) cell lines, as presented in Figure 1A. (B) MCT1 expression in native BT20 cells and BT20 cells transfected with siRNA against MCT1 (siMCT1), 1 and 2 days post-transfection, as presented in Figure 1G. (C) Western blots for the assessment of MCT1 and MCT4 expression in the TNBC cell lines, BT20 and MDA-MB-231.

**Figure S2: RNA expression data for breast cancer cell lines.** RNA expression of isoforms MCT1, 2 and 4, encoded by SLC16A1, 7 and 3, respectively were plotted for breast cancer cell lines. The RNA expression data for each isoform were derived from the Cancer Cell Line Encyclopedia (CCLE) database, expressed as log_2_(Fold Change) and plotted using Prism (GraphPad, CA, USA).

**Figure S3:** Bromide level (^78^Br and ^80^Br) normalized to control measured by LC-MS/MS in lysates from BT20 cells and BT20 cells transfected with siRNA targeted against MCT1 (siMCT1-BT20) or scramble siRNA (sc-BT20) (n=6). 3BP uptake is significantly different between BT20 and siMCT1-BT20 at all time points, but no differences evident when comparing BT20 to the scrambled control cells. Error bars represent SD, **** represents *P*<0.001 as determined by multiple t-tests.

**Figure S4:** **Effect of sub-toxic concentration of 3BP on the extracellular acidification rate (ECAR), oxygen consumption rate (OCR) and the OCR/ECAR ratio of TNBC cells.** BT20 and MDA-MB-231 cells were treated with 20 µM 3BP for 24 h in DMEM prior to measurement of the OCR and ECAR. Statistical significance was calculated using ‘unmatched’ two-way ANOVA, with the *P* value corrected for multiple comparisons using the Sidak test. Multiplicity adjusted *P* value is reported. N=4. Ns *P*>0.05, **P*<0.05. Error bars represent the standard deviation from the mean.
